# Effects of the Energy-Adjusted Dietary Inflammation Index During Pregnancy on Prenatal Depression: The Mediating Effect of Sleep Quality

**DOI:** 10.3390/nu17071197

**Published:** 2025-03-29

**Authors:** Yuehan Yuan, Jingjing Xu, Qian Lin, Jing Deng, Yunfeng Pan, Jihua Chen

**Affiliations:** 1 Department of Nutrition Science and Food Hygiene, Xiangya School of Public Health, Central South University, No. 172, Tongzipo Road, Yuelu District, Changsha 410013, China; 2 Xiangtan City Center for Disease Prevention and Control, 304 East Yangjiang Road, Yuhu District, Xiangtan 411100, China; 3 Department of Epidemiology and Health Statistics, Xiangya School of Public Health, Central South University, No. 172, Tongzipo Road, Yuelu District, Changsha 410013, China; 4 Department of Food Safety Supervision, Hunan Provincial Center for Disease Control and Prevetion, No. 450, Section 1, Furong Middle Road, Kaifu District, Changsha 410153, China

**Keywords:** antenatal depression, energy-adjusted dietary inflammatory index, mediating effect, sleep quality

## Abstract

(1) Background: Prenatal depression is prevalent and can adversely affect maternal and infant health. This study aimed to analyze the relationship between the energy-adjusted dietary inflammatory index (E-DII) and prenatal depression, as well as to explore the mediating effect of sleep quality. (2) Methods: In this cross-sectional study, a total of 749 pregnant women were enrolled. The E-DII scores were evaluated using semi-quantitative Food Frequency Questionnaires (FFQ); the Edinburgh Postpartum Depression Scale (EPDS) was used to measure depression levels; and the Pittsburgh Sleep Quality Index (PSQI) to evaluate the sleep quality of pregnant women. Binary logistic regression analysis was employed to analyze the relationships of E-DII with prenatal depression, of E-DII with sleep quality, and of sleep quality with prenatal depression. The bootstrap approach was employed to investigate the mediating effect of sleep quality. (3) Results: Findings indicated that a higher E-DII score was significantly associated with an increased risk of prenatal depression compared to the lowest score, and this association still existed after adjusting for sleep quality. In addition, the lowest E-DII score was also associated with a lower risk of poor sleep quality. Sleep quality played a partial mediating role in the association between E-DII and prenatal depression, and the proportion of the mediation effect relative to the total effect was 34.30%. (4) Conclusions: Sleep quality partially mediated the association between E-DII and prenatal depression. Close monitoring and proactive improvement of sleep quality among pregnant women following a pro-inflammatory diet may help reduce the risk of developing prenatal depression.

## 1. Introduction

Pregnancy involves significant physiological adaptations and psychosocial uncertainties that predispose women to emotional disturbances, including anxiety, persistent fears, and frustration during gestation and postpartum transition [1]. Unmanaged psychological distress in this period may progress to depression, clinically defined by the American College of Obstetricians and Gynecologists (ACOG) as depressive episodes occurring during pregnancy (prenatal depression) or within 12 months post-delivery (postpartum depression) [2]. Although the research field focuses more on postpartum depression, a recent study suggests the significance of prenatal depression since it might be a principal predictor of postpartum depression [3]. A cohort study also demonstrates an 11-fold increased risk of postpartum mental disorders in women with antenatal psychological distress [4]. Prenatal depression further correlates with adverse maternal–fetal outcomes, underscoring its clinical significance [5,6,7].

Dietary inflammation correlates with a variety of pregnancy complications, including gut dysbiosis, decreased bone density, and hyperemesis gravidarum [8,9,10]. Emerging research focuses on the impact of dietary inflammatory potential on mental health, quantified using the dietary inflammatory index (DII). DII was initially developed by Cavicchia, a public health expert at the University of South Carolina, in 2009 [11] and refined in 2014 [12]. DII assesses the dietary inflammatory potential by examining the combined effects of 45 food parameters on six inflammatory biomarkers, including interleukin-6 (IL-6), C-reactive protein (CRP), interleukin-1β (IL-1β), interleukin-4 (IL-4), interleukin-10 (IL-10), and tumor necrosis factor-α (TNF-α) [13], demonstrating strong concordance with clinical inflammation markers [14]. Several studies indicate a significant association between DII scores and depression. For instance, the Mediterranean dietary pattern, characterized by a high intake of fruits, vegetables, olive oil, whole grains, and fish, is associated with lower levels of inflammation and is regarded as an anti-inflammatory diet [15]. Results from randomized controlled trials (RCT) indicate that interventions based on the Mediterranean dietary practices can enhance mental health outcomes among patients suffering from depression [16]. Data from the United States National Health and Nutrition Examination Survey (NHANES) reveal a 2.26-fold increased risk for depression when comparing individuals in the highest versus lowest DII quintiles (OR: 2.26, 95%CI: 1.60–3.20) [17]. A meta-analysis further supports this association, comparing extreme inflammatory diet groups (OR: 1.45, 95%CI: 1.30–1.62) [18].

Pro-inflammatory diets demonstrate significant associations with depression, though underlying mechanisms remain incompletely characterized. Current evidence implicates sleep quality as a potential mediator. Numerous studies have established a correlation between inflammatory diets and diminished sleep quality. Prior research has indicated that sleep disturbances are associated with imbalances in the body’s inflammatory levels, whereby chronic low-grade inflammation contributes to sleep deprivation and compromised sleep quality [19,20]. An Italian cohort study reveals an association between a pro-inflammatory diet and impaired sleep quality [21]. RCT evidence further agreed with the therapeutic potential of the Mediterranean dietary interventions, showing clinically significant improvements in sleep quality versus usual care (mean PSQI 7.0 ± 0.2 SE vs. 7.9 ± 0.2 SE, *p* = 0.001) [22]. Sleep quality strongly influences mood in pregnant women. with poor sleep significantly increasing risks for anxiety and depression. A meta-analysis shows that pregnant individuals with poor sleep have 3.72 times higher depression risk than good sleepers [23]. This pattern is further aligned with a prospective cohort study involving 1152 Chinese pregnant women, where poor sleepers experience 3.3 times greater prenatal depression likelihood (OR: 3.30, 95% CI: 2.41–4.51) [24].

In summary, the mechanisms linking pro-inflammatory diets to prenatal depression require further investigation. This study aims to explore the mediating effect of sleep quality between these two factors. We propose that pro-inflammatory diets increase the risk of prenatal depression, with sleep quality mediating this effect. These findings could guide prenatal nutrition planning and sleep improvement strategies to help prevent pregnancy-related depression.

## 2. Materials and Methods

### 2.1. Study Design and Participants

**Figure 1:**
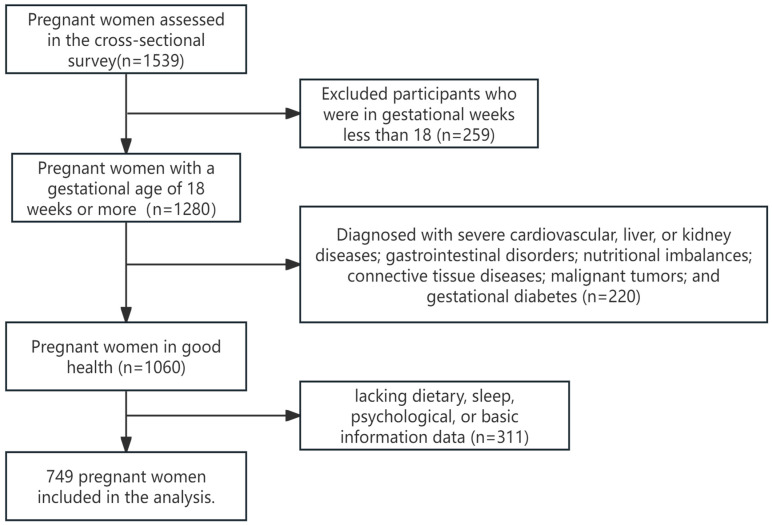
Study participant selection flow chart.

The pregnant women were recruited in the obstetric outpatient departments of Hunan Provincial Maternal and Child Health Hospital and Liling City Maternal and Child Health Hospital from January 2021 to January 2023. All assessments were conducted in person by the investigators. The inclusion criteria were as follows: (1) gestational age of 18 weeks or greater; (2) voluntary participation in the study with signed informed consent after being adequately informed about the project. The exclusion criteria included the following: (1) patients with severe cardiovascular, liver, or kidney diseases; gastrointestinal disorders; nutritional imbalances; connective tissue diseases; malignant tumors; and gestational diabetes; (2) pregnant women demonstrating abnormal expression or comprehension abilities; and (3) missing dietary, sleep, psychological, or basic information data (Figure 1).

The Ethics Committee of Tongji Medical College, Huazhong University of Science and Technology Institutional Review Board, approved the study protocol, and all participants provided written informed consent. The Ethical Approval Number for this research is Kuai202153.

### 2.2. Dietary Assessment

A semi-quantitative Food Frequency Questionnaire (FFQ) was used to assess the dietary intake of pregnant women. FFQ consists of three parts: food categories, consumption frequency, and the average portion size for each consumption; there were 11 food categories representing 43 food items, including cereals and potatoes, meat, aquatic products, eggs, milk, soybeans and their products, vegetables, fruits, snacks, nuts, and water/drinks. For each food item in the FFQ, respondents were asked how often they had consumed it and the average amount of each intake of various foods in the past 1 month. During the questioning, a special quantitative food model was used to help the study subjects recall the amounts of various foods they had ingested.

### 2.3. Sleep Quality Assessment

Sleep quality in pregnant women was assessed using the Pittsburgh Sleep Quality Index (PSQI). The scale consists of 19 self-rating items and 5 other scoring items. The scale includes seven dimensions: subjective sleep quality, sleep duration, sleep latency, sleep efficiency, sleep disorders, hypnotic drugs, and daytime function. Each component was scored according to the 0~3 level. The PSQI is the sum of the seven dimensions of the score, with higher scores indicating worse sleep quality, and PSQI scores ≥ 5 were defined as poor sleep quality according to previous studies [25,26]. The Chinese version of the PSQI demonstrated good reliability (Cronbach’s α: 0.82–0.83) [27].

### 2.4. Measurement of Depression

The Edinburgh Postnatal Depression Scale (EPDS) was used to assess the mental health status of pregnant women. This scale has been proven to be applicable for screening prenatal depression and consists of 10 items related to mood [28], enjoyment, self-blame, anxiety, fear, insomnia, coping, sadness, crying, and self-harm. Responses to each item are divided into four levels, reflecting different degrees of symptom severity. The main statistical indicator of EPDS is the total score, which is the sum of the scores of the 10 items, ranging from 0 to 30. Based on previous studies, we defined prenatal depression as an EPDS score of ≥10 [29]. The Chinese version of the EPDS demonstrated good reliability (Cronbach’s α: 0.78) [30].

### 2.5. Covariate Assessment

Covariates were obtained through questionnaires, including sociodemographic information (maternal age, occupation, family income per capita, and education level), physiological information (height and pre-pregnancy weight), maternal and maternal information (gestational age, gravidity, parity, mode of conception, and number of fetuses), behavior, and lifestyle (exercise habits and passive smoking). The pre-pregnancy body mass index (BMI) was divided into underweight (<18.5 kg/m^2^), normal weight (18.5~23.9 kg/m^2^), overweight (24.0~27.9 kg/m^2^), and obesity (≥28.0 kg/m^2^) according to China’s classification [31]. Passive smoking was defined as passive smoking for over 15 min for at least one day per week in the past month. The second trimester was defined as gestational week < 28, and the third trimester was defined as gestational week ≥ 28 [32].

### 2.6. Statistical Analysis

#### 2.6.1. E-DII Calculation

E-DII was used to rank the dietary inflammatory potential of pregnant women, and the specific calculation method was as follows:

In this study, MATLAB R2019a was used to matrix the average daily intake of food and the nutrient content of food to obtain the average daily intake of various nutrients of the study subjects. The dietary inflammatory potential is reflected by DII. Nevertheless, owing to the variations in body shape, physical activity, and metabolic efficiency, the energy intake of pregnant women varies. Hence, further energy adjustments to dietary nutrients should be conducted to transform DII into E-DII so as to minimize the impact of energy on nutrient intake as much as possible. In this study, E-DII was obtained by converting the daily intake and global average intake of all nutrients used in the calculation into the intake per 1000 Kcal of energy [33]. The development of the original DII includes 45 dietary parameters. Cultural dietary patterns limited perinatal women’s consumption of specific foods in China, requiring E-DII calculation to include 21 nutrients based on a previous study [34], and they were folic acid, saturated fatty acids, monounsaturated fatty acids, polyunsaturated fatty acids, carotene, energy, protein, fat, carbohydrates, dietary fiber, cholesterol, vitamin A (retinol equivalent), vitamin B1, vitamin B2, niacin, vitamin C, vitamin E, magnesium, iron, zinc, and selenium.

Z score = (daily intake of this kind of dietary ingredient or nutrient − this kind of dietary ingredient or the global average per capita daily intake of nutrients)/the SD of the global average per capita daily intake of this dietary ingredient or nutrient

Z score1 = Z score → (converted to a percentile score) × 2 − 1

E-DII = ΣZ score1 × the inflammatory effect score of each dietary component

The centralized Z-value of each nutrient or food is multiplied by the corresponding inflammatory effect score to obtain the E-DII score of each nutrient or food of the individual, and the E-DII score of each nutrient or food of the individual is summed to obtain the “total E-DII score”. The higher the E-DII value, the greater the pro-inflammatory potential of the diet. In group comparison, the study of all pregnant women’s E-DII scores from small to large, according to the tertiles method, divides E-DII into three groups, respectively: Q1 group (the most anti-inflammatory diet group), Q2 group (middle group), and Q3 (the most pro-inflammatory diet group).

#### 2.6.2. Data Analysis

Statistical description, comparison, and regression analysis were performed with SPSS25.0 (IBM Corp., Armonk, NY, USA). The quantitative data were transformed into qualitative data and expressed as a percentage, including age, pre-pregnancy BMI, gravidity, parity, PSQI score, and E-DII score. The prevalence rate of prenatal depression in each group was compared by the chi-square method. The direct effects of the E-DII and sleep quality on prenatal depression, as well as the impact of E-DII on sleep quality, were evaluated using three binary logistic regression models. The odds ratios (ORs) and 95% confidence intervals (CIs) for the associations between E-DII and prenatal depression, E-DII and sleep quality, and sleep quality and prenatal depression risk were estimated. The continuous variables of E-DII, PSQI score, and EPDS score were used for mediation analysis. Mediation hypotheses of 7 components of the PSQI questionnaire and the PSQI total score on the relationship between E-DII and prenatal depression were performed using the bootstrap method with 749 samples to calculate confidence intervals (95%). The method is non-parametric, does not rely on normal distribution assumptions, and is easy to understand and implement. The results were statistically significant with *p* < 0.05. An indirect effect was considered significant when the confidence interval did not include zero.

## 3. Results

### 3.1. Basic Characteristics of the Participants

This study included a total of 749 pregnant women, with an average age of 30.46 ± 4.16 years, an average gestational week of 27.34 ± 5.75, and an average pre-pregnancy BMI of 21.56 ± 3.41. The demographic characteristics and sleep quality of pregnant women grouped and classified by E-DII scores were shown in Supplementary Materials (Table S1), which showed that pregnant women with low education level and income and no exercise habits were more inclined to a pro-inflammatory diet. Table 1 describes the demographic characteristics, sleep quality, and E-DII levels of pregnant women grouped according to prenatal depression. The results showed that 40.85% of pregnant women had prenatal depression, with 60.08% experiencing poor sleep quality. The participants with the following characteristics exhibited a higher likelihood of developing prenatal depression, including being ≤24 years old, having a bachelor’s degree or lower, having a family income per capita < 5000 CNY/month, being unemployed, having poor sleep quality, and being in the highest tertile in the pro-inflammatory diet group (Q3). The differences in nutrient intake between depressed and non-depressed groups were shown in Supplementary Materials (Table S2). The findings indicated that the non-depressed group exhibited a higher intake of folic acid, carotene, dietary fiber, vitamin A, vitamin C, and magnesium in comparison to the depressed group. Conversely, their carbohydrate intake was lower.

### 3.2. Relationship Between E-DII, Sleep Quality, and Prenatal Depression During Pregnancy

Table 2 shows the relationship between the E-DII, sleep quality, and prenatal depression, with prenatal depression and sleep quality serving as dependent variables. Model 1 represents a binary logistic regression analysis assessing the association between prenatal depression and E-DII. Model 2 is a binary logistic regression analysis that evaluates the relationship between prenatal depression and E-DII while also controlling sleep quality. Model 3 conducted a binary logistic regression analysis to explore the connection between sleep quality and E-DII. All models were adjusted for age, pre-pregnancy BMI, pregnancy stage, education level, family income per capita, mode of conception, number of fetuses, gravidity, parity, passive smoking exposure, exercise habits, and employment status.

In model 1, the results of regression analysis showed a significant correlation between E-DII and prenatal depression, Q2 compared to Q1 (OR: 1.607, 95%CI: 1.087–2.375) and Q3 compared to Q1 (OR: 2.640, 95%CI: 1.789–3.897). Model 2 showed that there was no statistically significant difference in the risk of prenatal depression between the Q2 and Q1 groups after adjusting for sleep quality, but Q3 was associated with an increased risk of prenatal depression compared with the Q1 group (OR: 1.988, 95%CI: 1.323–2.986), indicating that there was a partial mediating effect on sleep quality. Model 3 showed the lowest E-DII score was also associated with a lower risk of poor sleep quality (OR: 0.314, 95%CI: 0.211–0.467).

### 3.3. The Mediating Effect of Sleep Quality in the Association Between E-DII and Prenatal Depression

**Figure 2:**
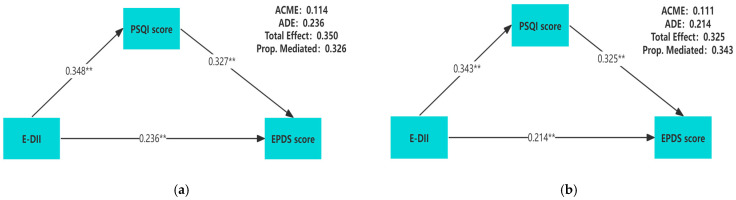
Unadjusted for any covariate (**a**); adjusted for gestational weeks, age, pre-pregnancy BMI, education level, family income per capita, gravidity, parity, exercise habits, and employment status (**b**). The mediation model of E-DII, sleep quality, and prenatal depression during pregnancy. All of the pathway coefficients are standardized. ** *p* < 0.01.

Table 3 showed the relative total, direct, and indirect effects for the mediating role of sleep quality on the relationship between E-DII and prenatal depression in mediation models. To better describe the correlation among these three variables, we conducted a mediation analysis using the original values of E-DII, PSQI scores, and EPDS scores. A significant mediating effect is indicated if the confidence interval for the indirect effect does not include 0 at the corresponding confidence level. Our mediation hypothesis was confirmed because bootstrapping revealed significant relative indirect effects for depression (ACME = 0.111, 95%CI: 0.088–0.137), indicating that sleep quality mediated the association between E-DII and depression. In addition, most sleep components also had mediating effects, such as subjective sleep quality (ACME = 0.051, 95%CI: 0.035–0.073), sleep latency (ACME = 0.036, 95%CI: 0.022–0.053), sleep duration (ACME = 0.025, 95% CI: 0.013–0.041), sleep efficiency (ACME = 0.020, 95%CI: 0.009–0.033), sleep disturbance (ACME = 0.063, 95%CI: 0.044–0.088), and daytime dysfunction (ACME = 0.059, 95%CI: 0.041–0.081). The mediation model was constructed by using the PSQI score as the mediator, and the results showed that regardless of whether the covariates are adjusted, there is a pairwise positive correlation among E-DII, PSQI scores, and EPDS scores (*p* < 0.01) (Figure 2).

## 4. Discussion

In this cross-sectional study, we investigated the association between E-DII and prenatal depression during pregnancy, as well as the mediating effect of sleep quality. The findings indicated that the risk of prenatal depression was significantly reduced when pregnant women transitioned from a highly pro-inflammatory diet (highest E-DII tertile, Q3) to a predominantly anti-inflammatory diet (lowest E-DII tertile, Q1). Furthermore, sleep quality was identified as a mediating factor in this relationship.

This study found that 40.85% of pregnant women experienced prenatal depression. Prior studies using the EPDS reported prenatal depression prevalence in Chinese pregnant women ranging from 16% to 65% [35,36,37]. Thus, while the prevalence observed here is relatively high, it aligns with previously reported ranges. Younger maternal age and lower socioeconomic status significantly predicted prenatal depression risk. Younger mothers, often first-time parents, demonstrated reduced psychological and financial preparedness for pregnancy. Lower-income families faced heightened financial stressors during gestation [38], exacerbating depression susceptibility. These populations require prioritized depression screening. Notably, socioeconomically vulnerable groups (lower education/income and physical inactivity) showed stronger adherence to inflammatory dietary patterns, necessitating targeted dietary monitoring and nutrition counseling during prenatal visits.

Pro-inflammatory dietary patterns demonstrated a significant association with prenatal depression. Individuals in the most pro-inflammatory diet group exhibited a two-fold higher risk compared to those in the most anti-inflammatory diet group, and the elevated risk persisted after adjusting for sleep quality. These findings aligned with previous research, which indicated that anti-inflammatory diets reduced nearly half of the risk of postpartum depression compared to the most pro-inflammatory diet group in China [34]. A pro-inflammatory diet elevated the level of inflammatory biomarkers and might trigger the onset of depression [39,40]. On the contrary, anti-inflammatory components, such as flavonols and omega-3 fatty acids, were associated with enhanced expression of neurotrophic factors, which mitigated depression by promoting synaptic growth among serotonin neurons in the brain [41,42,43].

Mediation analysis showed sleep quality explained 34.3% of E-DII’s effect on prenatal depression. Pro-inflammatory diets, which had higher E-DII, were consistently linked to worse sleep quality, aligning with previous studies. NHANES data found those with higher intake of inflammatory foods had an elevated risk of short sleep (<6 h/night; OR = 1.40, 95%CI: 1.21–1.61) and sleep problems (OR = 1.14, 95%CI: 1.02–1.27) compared to anti-inflammatory diet consumers [44]. An anti-inflammatory diet as a nonpharmacologic treatment was found to improve sleep quality in adults [45]. While sleep deprivation increased the risk of depression [46], treating insomnia alleviated depressive symptoms [47]. Our finding showed a decreased risk of prenatal depression among pregnant women with good sleep quality compared to those with poor sleep quality (OR: 0.268, 95%CI: 0.189–0.380), which was consistent with the results of several previous studies. Kalmbach et al. demonstrated significantly higher rates of depression and suicidal ideation among pregnant women with insomnia versus those without [48]. Lin et al. further identified associations between poor sleep patterns (e.g., late bedtime and sleep difficulties) and antenatal anxiety/depression during the COVID-19 pandemic [49]. Potential mechanisms involve sleep-related inflammation: poor sleep quality correlates with elevated pro-inflammatory cytokines (e.g., IL-6 and CRP) [50], which may disrupt neural circuits regulating mood, thereby increasing depression risk.

Our findings suggested that anti-inflammatory diets might mitigate systemic inflammation, subsequently improving sleep quality in pregnant women. Enhanced sleep protects emotional regulation, potentially reducing prenatal depression risk. Existing studies consistently indicate that high-quality, nutritious diets are effective in improving mental health and mood [51,52,53], particularly those emphasizing whole grains, plant-based foods, and lean proteins while minimizing refined carbohydrates [54]. Notably, the prenatal depression group showed a significantly lower intake of six anti-inflammatory nutrients, including folate, carotenoids, fiber, vitamins A/C, and magnesium, alongside higher pro-inflammatory carbohydrate consumption. An Irish cohort study corroborated the importance of fiber, magnesium, and B vitamins for gestational mental health [55], supporting targeted micronutrient supplementation. Clinically, close monitoring and proactive improvement of sleep quality among pregnant women following a pro-inflammatory diet may help reduce the risk of developing prenatal depression.

There was limited literature exploring the relationship between E-DII during pregnancy with both prenatal depression and sleep quality. To our knowledge, this is the first study investigating the sleep quality and its association between E-DII and prenatal depression with a large-scale sample size and comprehensive adjustment for key covariates such as maternal age, BMI, and socioeconomic status. The major limitation was its cross-sectional design, which precluded causal inference among E-DII, sleep quality, and depression. In addition, the dietary recall lasted for one month, which might introduce reporting bias. Lastly, the single-region recruitment restricted population generalizability. Future prospective cohort studies should validate these associations while exploring additional biological and behavioral mediators.

## 5. Conclusions

In conclusion, this study identified pro-inflammatory dietary patterns and poor sleep quality as risk factors for prenatal depression in Chinese pregnant women. Sleep quality served as a mediator between E-DII and prenatal depression. Moving forward, it is essential to prioritize improving sleep quality for pregnant women following a pro-inflammatory diet and to incorporate dietary and sleep management into routine prenatal care. By integrating nutritional counseling, sleep monitoring, and psychological support, we can develop systematic prevention strategies to reduce the risk of prenatal depression.

## Figures and Tables

**Table 1:** Sample characteristics of pregnant women and univariate analysis.

Characteristics	Population	EPDS Score < 10	EPDS Score ≥ 10	*p* Value ^1^
	*n* (%)	*n* (%)	*n* (%)	
N	749 (100)	443 (59.15)	306 (40.85)	
Age, years				0.002 **
≤24	46 (6.14)	15 (32.61)	31 (67.39)	
25~29	263 (35.11)	163 (61.98)	100 (38.02)	
30~34	329 (43.93)	201 (61.09)	128 (38.91)	
≥35	111 (14.82)	64 (57.66)	47 (42.34)	
Pregnancy stage				0.978
Middle Pregnancy	423 (56.48)	250 (59.10)	173 (40.90)	
Late Pregnancy	326 (43.52)	193 (59.20)	133 (40.80)	
Pre-pregnancy BMI, kg/m^2^				0.214
<18.5	110 (14.69)	71 (64.55)	39 (35.45)	
18.5~23.9	497 (66.36)	298 (59.96)	199 (40.04)	
24.0~27.9	109 (14.55)	56 (51.38)	53 (48.62)	
≥28	33 (4.41)	18 (54.55)	15 (45.45)	
Education level				<0.001 **
Below bachelor’s degree	370 (49.40)	192 (51.89)	178 (48.11)	
Bachelor’s degree	304 (40.59)	197 (64.80)	107 (35.20)	
Postgraduate and above	75 (10.01)	54 (72.00)	21 (28.00)	
Family income per capita, CNY/ month				<0.001 **
<5000	201 (26.84)	90 (44.78)	111 (55.22)	
5000~9999	284 (37.92)	173 (60.92)	111 (39.08)	
≥10,000	264 (35.25)	180 (68.18)	84 (31.82)	
Mode of conception				0.485
Natural conception	671 (89.59)	394 (58.72)	277 (41.28)	
Assisted reproduction	78 (10.41)	49 (62.82)	29 (37.18)	
Number of fetuses				0.634
1	719 (96.00)	424 (58.97)	295 (41.03)	
2	30 (4.01)	19 (63.33)	11 (36.67)	
Gravidity				0.273
1	336 (44.86)	203 (60.42)	133 (39.58)	
2	211 (28.17)	130 (61.61)	81 (38.39)	
≥3	202 (26.97)	110 (54.46)	92 (45.54)	
Parity				0.082
0	446 (59.55)	278 (62.33)	168 (37.67)	
1	264 (35.25)	142 (53.79)	122 (46.21)	
2	39 (5.21)	23 (58.97)	16 (41.03)	
Passive smoking				0.281
Yes	138 (18.42)	76 (55.07)	62 (44.93)	
No	611 (81.58)	367 (60.07)	244 (39.93)	
Exercise habits				0.161
Yes	378 (50.47)	233 (61.64)	145 (38.36)	
No	371 (49.53)	210 (56.60)	161 (43.40)	
Employment status				<0.001 **
Employed	536 (71.56)	338 (63.06)	198 (36.94)	
Unemployed	213 (28.44)	105 (49.30)	108 (50.70)	
Sleep quality				<0.001 **
Good sleep quality	299 (39.92)	230 (76.92)	69 (23.08)	
Poor sleep quality	450 (60.08)	213 (47.33)	237 (52.67)	
E-DII				<0.001 **
Q1	250 (33.38)	177 (70.80)	73 (29.20)	
Q2	250 (33.38)	151 (60.40)	99 (39.60)	
Q3	249 (33.24)	115 (46.18)	134 (53.82)	

E-DII, energy-adjusted dietary inflammatory index. ^1^ Chi-square test. ** *p* < 0.01.

**Table 2:** Binary logistic regression model for the relationship between E-DII, sleep quality, and prenatal depression.

Outcome Variable	Model 1: Prenatal Depression	Model 1: Prenatal Depression	Model 3: Sleep Quality
	OR, 95%CI	OR, 95%CI	OR, 95%CI
E-DII (reference: Q1)			
Q2	1.607 (1.087, 2.375) *	1.454 (0.968, 2.185)	0.701 (0.484, 1.017)
Q3	2.640 (1.789, 3.897) **	1.988 (1.323, 2.986) **	0.314 (0.211, 0.467) **
Sleep quality (reference : poor)			
Good	_	0.266 (0.186, 0.382) **	_
Age (reference: ≤24)			
25~29	0.392 (0.192, 0.799) *	0.342 (0.161, 0.728) **	0.862 (0.431, 1.725)
30~34	0.396 (0.193, 0.813) *	0.313 (0.146, 0.672) **	0.616 (0.304, 1.245)
≥35	0.474 (0.209, 1.077)	0.411 (0.173, 0.976) *	0.767 (0.342, 1.723)
Pre-pregnancy BMI (reference:<18.5) kg/m^2^			
18.5~23.9	1.443 (0.908, 2.291)	1.110 (0.680, 1.811)	0.406 (0.259, 0.636) **
24.0~27.9	1.843 (1.034, 3.286) *	1.562 (0.852, 2.866)	0.532 (0.302, 0.937) *
≥28	1.597 (0.695, 3.669)	1.306 (0.560, 3.044)	0.468 (0.205, 1.071)
Pregnancy stage (reference: middle Pregnancy)			
Late Pregnancy	1.104 (0.805, 1.515)	1.007 (0.724, 1.401)	0.731 (0.532, 1.005)
Education level (reference: Below bachelor’s degree)			
Bachelor’s degree	0.798 (0.548, 1.163)	0.717 (0.483, 1.064)	0.760 (0.520, 1.110)
Postgraduate and above	0.691 (0.375, 1.276)	0.565 (0.299, 1.066)	0.560 (0.308, 1.017)
Family income per capita, CNY/ month (reference: <5000)			
5000~9999	0.651 (0.436, 0.970) *	0.612 (0.402, 0.932) *	0.912 (0.602, 1.382)
≥10,000	0.554 (0.356, 0.862) **	0.564 (0.355, 0.896) *	1.243 (0.790, 1.955)
Mode of conception (reference: Natural conception) Assisted reproduction			
	0.972 (0.543, 1.740)	1.122 (0.608, 2.071)	1.608 (0.896, 2.885)
Number of fetuses (reference: 1)			
2	0.839 (0.355, 1.982)	0.757 (0.306, 1.870)	0.805 (0.340, 1.904)
Gravidity (reference: 1)			
2	0.671 (0.416, 1.081)	0.671 (0.408, 1.102)	1.100 (0.691, 1.753)
≥3	0.845 (0.488, 1.464)	0.834 (0.469, 1.484)	1.066 (0.615, 1.848)
Parity (reference: 0)			
1	1.416 (0.876, 2.289)	1.510 (0.914, 2.495)	1.078 (0.667, 1.744)
2	0.893 ()0.382, 2.089	0.882 (0.366, 2.123)	0.850 (0.353, 2.045)
Passive smoking (reference: No)			
Yes	1.071 (0.717, 1.600)	1.029 (0.678, 1.562)	0.850 (0.563, 1.283)
Exercise habits (reference: No)			
Yes	0.958 (0.698, 1.313)	1.010 (0.727, 1.402)	1.243 (0.905, 1.708)
Employment status (reference: Unemployed)			
Employed	0.811 (0.559, 1.177)	0.909 (0.615, 1.343)	1.523 (1.037, 2.253) *

E-DII, energy-adjusted dietary inflammatory index; Model 1: binary logistic regression analysis between prenatal depression and E-DII without adjusting for sleep quality; Model 2: binary logistic regression analysis between prenatal depression and E-DII adjusted by sleep quality; Model 3: binary logistic regression between sleep quality and E-DII. All models had been adjusted by age, pre-pregnancy BMI, pregnancy stage, education level, family income per capita, mode of conception, number of fetuses, gravidity, parity, passive smoking, exercise habits, and employment status. * *p* < 0.05, ** *p* < 0.01.

**Table 3:** Mediating model: the mediating role of sleep quality in the relationship between E-DII and prenatal depression.

Mediator Variable		*β*	*p* Value	95% CI
PSQI	ACME	0.111	<0.001 **	0.088–0.137
	ADE	0.214	<0.001 **	0.163–0.266
	Total Effect	0.325	<0.001 **	0.273–0.376
	Prop. Mediated	0.343		
Subjective Sleep Quality	ACME	0.051	<0.001 **	0.035–0.073
	ADE	0.274	<0.001 **	0.218–0.328
	Total Effect	0.325	<0.001 **	0.273–0.376
	Prop. Mediated	0.158		
Sleep Latency	ACME	0.036	<0.001 **	0.022–0.053
	ADE	0.290	<0.001 **	0.238–0.338
	Total Effect	0.325	<0.001 **	0.273–0.376
	Prop. Mediated	0.109		
Sleep Duration	ACME	0.025	0.002 **	0.013–0.041
	ADE	0.300	<0.001 **	0.246–0.353
	Total Effect	0.325	<0.001 **	0.273–0.376
	Prop. Mediated	0.078		
Sleep efficiency	ACME	0.020	0.007 **	0.009–0.033
	ADE	0.306	<0.001 **	0.255–0.357
	Total Effect	0.325	<0.001 **	0.273–0.376
	Prop. Mediated	0.060		
Sleep Disturbance	ACME	0.063	<0.001 **	0.044–0.088
	ADE	0.262	<0.001 **	0.209–0.317
	Total Effect	0.325	<0.001 **	0.273–0.376
	Prop. Mediated	0.194		
Used Sleep Medication	ACME	0.002	0.492	−0.001–0.012
	ADE	0.323	<0.001 **	0.271–0.373
	Total Effect	0.325	<0.001 **	0.273–0.376
	Prop. Mediated	0.007		
Daytime Dysfunction	ACME	0.059	<0.001 **	0.041–0.081
	ADE	0.266	<0.001 **	0.214–0.317
	Total Effect	0.325	<0.001 **	0.273–0.376
	Prop. Mediated	0.181		

ACME, average causal mediation effects (indirect effect); ADE, average direct effects; Prop. Mediated, the mediator variable explains the percentage of the association between E-DII and prenatal depression. ** *p* < 0.01.

## Data Availability

The data that support the findings of this study are not publicly available due to the data containing information that could compromise the participants’ privacy but are available from the corresponding author upon reasonable request.

## Supplementary Materials

The following supporting information can be downloaded at: https://www.mdpi.com/article/10.3390/nu17071197/s1. Table S1: Distribution of the E-DII levels across sociodemographics. Table S2: Comparison of nutrient intake in pregnant women with and without depression.

## Abbreviations

The following abbreviations are used in this manuscript:

ACOG	American College of Obstetricians and Gynecologists
DII	Dietary inflammatory index
NHANES	The United States National Health and Nutrition Examination Survey
FFQ	Food Frequency Questionnaire
PSQI	Pittsburgh Sleep Quality Index
EPDS	Edinburgh Postnatal Depression Scale
BMI	Body mass index
E-DII	Energy-adjusted dietary inflammatory index
ACEM	Average causal mediation effects
ADE	Average direct effects
OR	Odds ratio
CI	Confidence interval
IL-6	Interleukin-6
CRP	C-reactive protein
