# Supplementary material for: Effects of the Energy-Adjusted Dietary Inflammation Index During Pregnancy on Prenatal Depression: The Mediating Effect of Sleep Quality
Source: Nutrients. 2025 Mar 29;17(7):1197. doi: 10.3390/nu17071197 (PMC11990411; doi:10.3390/nu17071197)
Supplement: Supplementary file 1 [file nutrients-17-01197-s001.zip › nutrients-3550155-supplementary.pdf]

Effect of the energy-adjusted dietary inflammation index during pregnancy on prenatal depression: the mediating effect of sleep quality

Supplementary Materials:

Table S1： Distribution of the E-DII levels across sociodemographics

Characteristics	E-DII Tertiles			χ^2	*P* Value
	Q1 n（%）	Q2 n（%）	Q3 n（%）		
N	250	250	249		
Age, years				6.576	0.362
≤24	13（28.3）	13（28.3）	20（43.5）		
25~29	78（29.7）	97（36.9）	88（33.5）		
30~34	115（35.0）	107（32.5）	107（32.5）		
≥35	44（39.6）	33（29.7）	34（30.6）		
Pregnancy stage				1.692	0.423
Middle Pregnancy	133（31.4）	144（34.0）	146（34.5）		
Late Pregnancy	117（35.9）	106（32.5）	103（31.6）		
Pre-pregnancy BMI, kg/m^2				3.950	0.683
<18.5	31（28.2）	37（33.6）	42（38.2）		
18.5~23.9	167（33.6）	168（33.8）	162（32.6）		
24.0~27.9	40（36.7）	32（29.4）	37（33.9）		
≥28	12（36.4）	13（39.4）	8（24.2）		
Education level				12.007	**0.017**
Below bachelor's degree	115（31.1）	123（33.2）	132（35.7）		
Bachelor’s degree	97（31.9）	107（35.2）	100（32.9）		
Postgraduate and above	38（50.7）	20（26.7）	17（22.7）		
Family income per capita, yuan/ month				17.632	**0.001**
＜5000	48（23.9）	67（33.3）	86（42.8）		
5000~9999	103（36.3）	88（31.0）	93（32.7）		
≥10000	99（37.5）	95（36.0）	70（26.5）		
Mode of conception				0.326	0.850
Natural conception	222（33.1）	224（33.4）	225（33.5）		
Assisted	28（35.9）	26（33.3）	24（30.8）		

reproduction					
Number of fetuses				1.442	0.486
1	241（33.5）	237（33.0）	241（33.5）		
2	9（30.0）	13（13.3）	8（26.7）		
Gravidity				3.402	0.493
1	119（35.4）	104（31.0）	113（33.6）		
2	61（28.9）	78（37.0）	72（34.1）		
≥3	70（34.7）	68（33.7）	64（31.7）		
Parity				2.525	0.640
0	155（34.8）	152（34.1）	139（31.2）		
1	84（31.8）	84（31.8）	96（36.4）		
2	11（28.2）	14（35.9）	14（35.9）		
Passive smoking				2.831	0.243
Yes	38（27.5）	48（34.8）	52（37.7）		
No	212（34.7）	202（33.1）	197（32.2）		
`				10.440	**0.005**
Yes	146（38.9）	114（30.4）	115（30.7）		
No	104（27.8）	136（36.4）	134（35.8）		
Employment status				3.909	0.142
Employed	190（35.4）	176（32.8）	170（31.7）		
Unemployed	60（28.2）	74（34.7）	79（37.1）		

Table S2： Comparison of nutrient intake in pregnant women with and without depression

Nutrient	EPDS		*P* Value
	EPDS score＜10	EPDS score≥10	
Folic acid	523.58±230.27	464.35±210.96	**＜0.001**
Saturated fatty acids	14.58±6.20	14.91±6.98	0.487
Monounsaturated fatty acids	15.09±6.37	15.46±7.49	0.463
Polyunsaturated fatty acids	10.59±7.08	10.01±6.10	0.238
Carotene	7855.50±3986.75	6652.10±3637.79	**＜0.001**
Energy	1959.61±510.96	2024.08±546.58	0.099
Protein	91.27±28.37	92.16±33.06	0.692
Fat	51.00±20.08	51.83±22.11	0.592
Carbohydrates	291.78±87.58	304.50±90.53	**0.048**
Dietary fiber	18.15±7.41	16.75±6.98	**0.009**
Cholesterol	464.61±189.83	491.95±240.62	0.083
Vitamin A	1213.49±666.95	1111.76±666.78	**0.040**
Vitamin B1	1.25±0.36	1.24±0.36	0.565
Vitamin B2	1.39±0.45	1.36±0.489	0.377
Niacin	17.58±0.54	17.84±6.39	0.545
Vitamin C	208.82±93.00	188.47±91.63	**0.003**
Vitamin E	28.11±15.38	26.75±15.55	0.234
Magnesium	497.36±155.52	462.24±152.39	**0.002**
Iron	24.94±8.31	23.86±8.84	0.090
Zinc	14.78±4.85	14.67±5.56	0.771
Selenium	49.89±17.60	50.83±24.08	0.539
